# Factors Influencing on Problem Solving Ability of Nursing Students Experiencing Simulation Practice

**DOI:** 10.3390/ijerph191811744

**Published:** 2022-09-17

**Authors:** Hyun Hee Jo, Won Ju Hwang

**Affiliations:** 1 Department of Nursing, Hyejeon College, 19 Daehak 1-gil, Hongseong-eup, Hongseong-gun 32244, Korea; 2 College of Nursing Science, East-West Nursing Research Institute, Kyung Hee University, 26 Kyunghee-Daero, Dongdaemun-gu, Seoul 02447, Korea

**Keywords:** self-leadership, goal commitment, critical thinking disposition, problem-solving ability, simulation practice

## Abstract

It has become important for nurses to implement self-leadership and exercise critical thinking in problem-solving to address the health issues of patients. This has led to a need for nursing education programs in which nursing students learn to embrace self-leadership and self-evaluation approaches to develop their skills. Within 260 nursing undergraduates with experience in simulation practice as study subjects, a self-reporting survey was conducted on self-leadership, goal commitment, critical thinking, and problem-solving skills. An analysis was conducted using the SPSS/WIN 21.0 program. *t*-test and ANOVA were conducted to validate the difference between problem-solving abilities. Multiple regression was conducted to examine the impact of these variables on problem-solving skills. The variables of religion, satisfaction with major, goal commitment, and critical thinking were found to have a significant impact on problem-solving abilities. The results were as follows: critical thinking (β = 0.36, *p* < 0.05), goal commitment (β = 0.28, *p* < 0.05), and explanatory power of 41%. To improve the nursing undergraduates’ problem-solving abilities through simulation practice, there needs to be a method that supports them in setting goals with self-leadership and enhance goal commitment. The method also needs to support the development of their critical thinking and curiosity for questions deriving from experiencing diverse programs in order to deliver effective outcomes.

## 1. Introduction

### 1.1. Background

In recent nursing environments, complex and strategic practices are required, and the role of nurses is recognized for its importance. In order to effectively respond to the patients’ demands, it has become important for nurses to equip themselves with specialized skills [1,2].

Such change requires nurses to build not only a high level of knowledge and skills, but also self-leadership to actively solve problems with independence and autonomy, as well as critical thinking to set the best goals and to identify strategies and grounds for apply clinical judgment and decision [3,4]. To this effect, nursing education must provide a learning environment that reflects actual practices and supports students to ultimately equip themselves with problem-solving abilities by coming up with their own measures, as well as searching and collecting the required data in order to exercise leadership and make logical and critical decisions in various situations, thereby ultimately carrying out nursing at a technical level [5]. Problem-solving abilities are an essential quality and the most notable characteristic of nursing professionals, in which they utilize their knowledge, explore and arrange information, and use it to serve the intended purposes under complex and unpredictable circumstances [6]. However, the traditional top-down education systems have limitations in teaching such practical skills and are inadequate to prepare students for many challenges in the field [7]. For nursing graduates, attitudes that are active, autonomous, responsible, and such are required in clinical practice, and many of them face difficulties to meet the requirements as they have developed passive and dependent approaches during early school years centered on university entrance exams [8].

Insufficient training on practice at nursing colleges leads to the decline in clinical practice capability of new nurses after their graduation. To complement this, more universities are adopting a high-performance simulator to enhance their training on practice [7,9]. The training simulators provide benefits by reproducing simulated situations which are similar to actual clinical environments, motivating the learning of students and providing direct learning effects by having students engage in activities in simulated situations. They also provide additional learning effects through post-learning evaluation and a re-design process. Furthermore, it has been reported that such practice-oriented training provides benefits of enhancing students’ critical thinking skills, allowing them to experience the outcomes of their clinical intervention, and to share with other students and reflect on their experience through de-briefing [10]. In simulated situations, trainees must apply in a critical way the theories learned to address patient issues and set their priorities to make decisions in a self-directed manner [11,12]. If students lack self-leadership, which is underscored in the simulated training on practice, they may not be able to improve problem-solving skills the essential skill required for change [13,14].

Due to the characteristics of nursing training, the result of training is directly related to practice in the field, and this has led to more attention being given to the design of training courses involving skills development and training experience for nurses aiming to develop and promote their critical-thinking and problem-solving abilities [15,16]. However, there is an insufficient number of studies on the relationship between self-leadership, goal commitment, critical thinking, and problem-solving abilities in nursing students. Thereby, the study results may be used as basic data for developing and operating training programs to enhance nursing undergraduates’ problem-solving skills.

### 1.2. Study Purpose

The purpose of this study was to identify the relationship between self-leadership, goal commitment, critical thinking, and problem-solving abilities in nursing undergraduates who experienced simulation training on practice and the impact of these variables on problem-solving processes. This involved (1) investigating the relationship between self-leadership, goal commitment, critical thinking, and problem-solving abilities; and (2) identifying the impact of self-leadership, goal commitment, and critical thinking on problem-solving abilities.

## 2. Methodology

### 2.1. Study Design

This is a descriptive investigation study conducted to identify the relationship between self-leadership, goal commitment, critical thinking, and problem-solving abilities of nursing undergraduates with the experience of simulation practice training.

### 2.2. Sampling Strategy

The participants of this study consisted of nursing undergraduates (juniors and seniors) at 4 universities who received 30 to 60 h of simulated practice training. The training was provided in one of the following two ways; one day of simulated practice training during clinical practice or five-day simulated practice training. All universities were equipped with high-performance simulators and used them in practice training. Though equipment and materials used varied by subject, high-performance simulators and dummies were used for most subjects.

### 2.3. Ethical Considerations

For the bioethics and safety of study subjects, this study was conducted after deliberation by the Institutional Review Board of University and the approval (No: KHSIRB-14-059(EA)) was obtained. During study period, the guidelines of the IRB were complied with. The description of the study was provided to study subjects and the purpose and methods of the study were explained. The survey was handed out to be filled-out by subjects who agreed to participate and they were informed the data they provided would be used solely for the study’s purpose. Study subjects were provided with a small amount of compensation in appreciation of their participation.

### 2.4. Study Methods

(1) Demographics

General characteristics included university, age, grade, religion, personal relationships, academic grades, major satisfaction, leadership training experience, and need for leadership. The age groups were divided into two: subjects of 22 years of age or below and subjects of 23 years of age or above. The personal relationships were classified into average, good, and very good. The major satisfaction was classified into unsatisfactory, average, satisfactory, and highly satisfactory.

(2) Self-leadership

To measure the self-leadership of nursing undergraduates, RSLQ (Revised Self-Leadership Questionnaire) developed by Houghton and Neck [17] was used after being modified and complemented by Shin et al. [18] for Korean participants. It consists of 35 items under 3 main categories and 9 sub-categories. The Cronbach’s α by item ranged from 0.74 to 0.88 and the Cronbach’s α value in this study was between 0.72 and 0.85.

(3) Goal commitment

To measure the goal commitment of nursing undergraduates, the tool suggested by Klein et al. [19] was used. The tool consisted of 5 items measured with the 5-point Likert scale. The higher score meant a greater goal commitment. Items 1, 2, and 3 were reverse items. The Cronbach’s α was 0.83 and the value in this study was 0.75.

(4) Critical thinking disposition

To measure the critical thinking disposition of nursing undergraduates, the tool suggested by Yoon [3] was used. The tool consisted of a total of 27 items under 7 categories of intellectual eagerness/curiosity, prudence, self-confidence, systematicity, intellectual fairness, healthy skepticism, and objectivity. In the study by Yoon [3], the Cronbach’s α was 0.84 and the value in this study was 0.74.

(5) Problem-solving abilities

The Social Problem Solving Inventory-Revised (SPSI-R) modified by D’Zurilla and Maydeu-Olivares [20] and translated by Choi [21] were used to measure problem-solving abilities. SPSI-R consisted of two major scales to measure the problem-solving orientation and problem-solving skills, as well as 5 sub-categories of positive problem orientation, negative problem orientation, rational problem-solving, impulsive/careless style, and avoidance coping style. The Cronbach’s α was 0.78 and the value ranged from 0.68 to 0.91 in this study.

### 2.5. Data Collection Method

The study data were collected from 18 September 2014 to 4 November 2014. The data were collected from juniors and seniors with an experience of simulated practice training from nursing departments at 4 universities in Seoul, and upon agreement for participation. The surveys were then handed out, filled-in, and retrieved straight away. A total of 280 copies were distributed and 276 copies were retrieved. With 16 surveys with insincere responses excluded, a total of 260 copies were used for final analysis.

### 2.6. Data Analysis

The data collected were analyzed using SPSS/WIN 21.0 program (IBM Corp., Armonk, NY, USA). The variables of mistakes, average, and percentage were used to identify general characteristics. The difference in problem-solving abilities by general characteristic was validated through *t*-test and ANOVA with additional validation through Scheffe test and Fisher’s exact test. The relationship between the subjects’ self-leadership, goal commitment, critical thinking, and problem-solving abilities was analyzed using Pearson’s correlation coefficient. A multiple regression was used to identify the impact of these variables on problem-solving abilities.

## 3. Results

### 3.1. General Characteristics of Study Participants

The subjects of this study comprised 260 juniors and seniors in the nursing department with the experience of 30 to 60 h of simulated training on practice. The juniors accounted for 72.3% and the good personal relationships accounted for 56.5%. The major satisfaction was 55.4% and no leadership training experience was 61.9%. The results were as follows: self-leadership of 3.59 ± 0.71, goal commitment of 3.68 ± 0.57, critical thinking of 3.57 ± 0.57, and problem-solving abilities of 3.14 ± 0.58 (Table 1).

### 3.2. Problem-Solving Abilities According to General Characteristics

The difference in problem-solving abilities according to the general characteristics of the study subjects showed significance in grades, personal relationships, academic grades, and major satisfaction. The juniors (2.75 ± 0.62) showed higher numbers of impulsive style (t = 1.30, *p* = 0.003) than seniors (2.62 ± 0.46) in relation to grades. The seniors showed higher numbers of positive problem orientation (t = −0.55, *p* = 0.013) and rational problem-solving (t = 0.00, *p* = 0.039). There were significant differences in positive problem orientation (F = 5.68, *p* = 0.004) and negative problem orientation (F = 7.96, *p* < 0.001) in relation to personal relationships. The result of post-validation shows that subjects with “average (3.65 ± 0.53)” personal relationships showed a lower positive problem orientation than subjects with “very good (2.51 ± 0.72)” personal relationships. The subjects with “very good (2.51 ± 0.72)” personal relationships showed a lower negative problem orientation than subjects with “average (3.09 ± 0.66)” and “good (2.97 ± 0.71)” personal relationships. Subjects with higher academic grades showed a lower negative problem orientation (F = 3.77, *p* = 0.024), impulsive careless style (F = 3.78, *p* = 0.024), and evasive style (F = 4.65, *p* = 0.010) than subjects with lower academic grades. There was a significant difference in all sub-categories of problem-solving abilities in relation to major satisfaction. For positive problem orientation (F = 5.35, *p* < 0.001), subjects with “average (3.62 ± 0.48)” showed lower problem-solving abilities than subjects with “very satisfactory (4.10 ± 0.47)”. For negative problem orientation (F = 6.32, *p* < 0.001), subjects with “average (3.18 ± 0.68)” showed higher problem-solving abilities than subjects with “satisfactory (2.84 ± 0.69)” and “very satisfactory (2.59 ± 0.76)”. For rational problem solving (F = 5.10, *p* = 0.001), subjects with “very satisfactory (3.82 ± 0.36)” showed higher problem-solving abilities than subjects with “average (3.43 ± 0.40)” and “unsatisfactory (3.48 ± 0.69)”. For impulsive/careless style (F = 4.24, *p* = 0.002), subjects with “unsatisfactory (2.95 ± 0.50)” showed higher problem-solving abilities than subjects with “satisfactory (2.65 ± 0.51)” and “very satisfactory (2.45 ± 0.63)”. While there was a significant difference observed in relation to evasive style (F = 2.99 *p* = 0.019), no difference was observed in the post validation. (Table 2)

### 3.3. Correlation between Subjects’ Self-Leadership, Goal Commitment, Critical Thinking, and Problem-Solving Abilities

A Pearson’s correlation was used to identify the correlation between general characteristics, self-leadership, goal commitment, critical thinking, and problem-solving abilities of nursing undergraduates with the experience of simulated practice training and the result shows that there was a positive correlation between problem-solving abilities and the variables of critical thinking (r = 0.54, *p* < 0.01), goal commitment (r = 0.47, *p* < 0.01), and self-leadership (r = 0.43, *p* < 0.05). For general characteristics, a positive correlation was observed in major satisfaction (r = 0.29, *p* < 0.01) and personal relationships (r = 0.21, *p* < 0.01; Table 3).

### 3.4. Influential Factors on Problem-Solving Abilities of Nursing Undergraduates with the Experience of Simulated Practice Training

To identify the influential factors on the problem-solving abilities of nursing undergraduates with the experience of simulated practice training, the variables that showed significance difference of *p* < 0.5 in relation to the subjects’ general characteristics were designated as potential influential factors. These variables were self-leadership, goal commitment, critical thinking, age, religion, personal relationships, major satisfaction, and leadership training experience. A multiple regression analysis was conducted and the results were as follows: For the analysis, the general characteristics of the subjects’ age, having or not having a religious belief, having a good or not good personal relationships, and being satisfied or unsatisfied about the major were processed as dummy-coded variables. First, as a result of testing the assumptions of regression analysis, it was found that all of them were satisfied.

Having a religious belief, major satisfaction, goal commitment, and critical thinking were observed to be significant influential factors on problem-solving abilities. The results were as follows: critical thinking (β = 0.36) and goal commitment (β = 0.28). Self-leadership, age, personal relationships, and leadership training experience showed no significance, with the value of *p* > 0.05. The regression equation used to analyze the influential factors showed the following results: R = 0.65, R^2^ = 0.42, modified R^2^ = 0.41, F = 23.17, *p* < 0.01, and Durbin–Watson = 1.97. The Durbin–Watson value was 1.97 and close to 2, ensuring the independence of error, and the explanatory power was 41%. The tolerance limit was 1 or below and the variance inflation factor (VIF) was lower than 10, showing no problem for multi-collinearity (Table 4). Next, as a result of analyzing influence using Cook’s D statistics, there was no more than 1.0 discrete in 260 subjects. Following a result of residual analysis, linearity of the model, normality of the error term, and homoscedasticity were confirmed.

## 4. Discussion

Nursing education and training must provide a learning environment that reflects actual practice and enables students to set up alternative measures to solve learning problems, search and obtain required data, and equip themselves with problem-solving abilities. However, the traditional top-down education systems have limitations in teaching such practical skills and are inadequate to prepare students for many challenges in the field [7]. This study aimed to analyze the relationship between the self-leadership, goal commitment, critical thinking, and problem-solving abilities of nursing undergraduates with the experience of simulated practice training and to obtain basic data to improve the quality of simulated practice training.

The results of the study are similar to the previous studies; an average mean of 3.14 points for problem-solving skills, 3.36 points for problem-solving orientation, and 2.98 points for problem-solving abilities [22]. This suggests that nursing undergraduates evaluated their problem-solving abilities to be of intermediate level and perceived themselves as an intermediate-level problem solver. For sub-categories, the positive problem orientation showed the highest point, followed by rational problem-solving and evasive style. This implies that nursing undergraduates showed a comparatively positive emotional state when faced with problems, but had a tendency to evade problems for as long as possible rather than confront them. According to the study by Cha et al. [22], nursing undergraduates who believed they were ineffective problem-solvers tended to imagine more hardships than there actually were, when faced with problems. To address this problem, the study also stressed out the need for measures to support nursing undergraduates to experience various situations and respond in objective, rational, and cognitive manners. Problem-solving abilities refer to a process of making decisions using effective problem-solving strategies based on one’s knowledge and are regarded as very important skills for professional nurses. A study by Yang [23] stated that the current nursing education and training courses provide content and methods that are not effective in teaching nursing undergraduates about the understanding or skills of the problem-solving process, and results in many undergraduates experiencing hardships when faced with various challenges in the clinical field. As insufficient training on practice leads to a decline in the clinical practice capability of new nurses after graduation; therefore, various teaching methods should be applied to enhance students’ problem-solving abilities and to enable nursing undergraduates to solve problems in a positive, active, and rational manner.

The difference in problem-solving abilities according to the general characteristics was partially significant for grades, personal relationships, and academic grades. The major satisfaction exhibited significant differences in all sub-categories. For positive problem orientation and rational problem-solving, the subjects with a greater major satisfaction showed a higher level of problem-solving skills. For negative problem orientation and impulsive/careless style, subjects with less major satisfaction showed a higher point. These results are consistent with the results of another study [24] on critical thinking disposition, problem-solving ability, and clinical competence of nursing students in that the subjects with a lower major satisfaction had lower problem-solving skills. This means that the subjects with a greater major satisfaction had more effective, intellectual, and creative problem-solving abilities than those with less major satisfaction. This is believed to be due to factors such as academic achievement, active attitude toward problem-solving, and such, according to their level of major satisfaction.

The influential factors for problem-solving abilities were goal commitment and critical thinking. Although the general characteristics of age, satisfaction with nursing major, and personal relationships showed correlations with problem-solving skills, no significant result was observed in the regression analysis. In a study by Aubé et al. [25], a virtual management project was implemented for undergraduates to examine the data provision and goal commitment. The results showed that subjects with a higher goal commitment had a higher problem-solving performance. The study also stated that subjects could develop actual capabilities required in the field only by having a deep understanding of situations and that they could acquire problem-solving abilities required in the field by committing to the situation not as an observer, but as a learner. Therefore, to enhance problem-solving skills, various programs should be developed and implemented to improve students’ goal commitment. In addition, team projects or action-based learning programs should be implemented to promote interactions between different learning programs and enhance students’ commitment to the study. In this way, the programs may enhance the problem-solving abilities of students by motivating them.

According to the study by Han and Park [26], greater critical thinking leads to higher problem-solving skills, which in turn enhances one’s confidence in problem-solving and improves the problem-solving skills. The study found that nursing undergraduates must develop critical thinking skills in order to explore problems in diverse aspects and seek solutions in a discreet manner. Training using simulators is an effective educational method as it motivates students’ learning and provides direct learning effects through simulated activities. Furthermore, it enhances the students’ critical abilities and allows them to experience the outcomes of their clinical intervention, as well as to share with other students and reflect on their experience through de-briefing [10].

Tucker et al. [27] stated that problems can be solved through a regular method based on guidelines and algorithms, and that such guidelines and algorithms are the result of such efforts. To develop problem-solving skills, which is an essential quality for nurses, one must develop a strong goal commitment and critical thinking abilities. To this effect, nursing undergraduates should be subjected to various nursing situations and be able to establish their own goals and commitment. They should also develop an accurate understanding of situations and critical thinking abilities to accurately identify, analyze, consolidate, and utilize data. To this effect, various simulated practice trainings, action-based learning, and problem-solving-oriented teaching programs, which support a natural learning process through a repetitive process, should be implemented to have learners actively engage in learning and develop a sense of commitment as well as critical thinking.

This study suggests that, while self-leadership showed no significant impact on problem-solving skills, it had impact on the problem-solving abilities of nursing undergraduates [28,29]. It was also suggested that self-leadership affected the goal commitment, which impacted the problem-solving abilities and improved it. This was due to the fact that not all nursing undergraduates exercised self-leadership and the level of self-leadership varied according to the individuals’ ability, environmental factors, function, and task structure [17]. Self-leadership involves a behavior strategy and cognitive strategy. Nursing students need to be committed to this process to solve problems and must put efforts to address problems on their own in order for their problem-solving abilities to develop. To enhance problem-solving skills, they must be committed to achieving their goals. Educational and training programs should be developed and implemented to provide nursing students with circumstances in which they are able to exercise self-leadership and in which they are encouraged to develop self-leadership skills.

This study holds significance in that it suggests the need for the development and implementation of various programs involving simulated practice training to enhance the problem-solving abilities of nursing undergraduates. It also suggests the need for measures to make students set their own goals and improve the level of their goal commitment, as well as the fact that the use of self-leadership may enhance the effectiveness of the process. Furthermore, it suggests the possible synergetic effects on developing critical thinking abilities by allowing students to build critical curiosity over questions they face by experiencing various programs. On the other hand, this study had limitations in that the scope of sample was limited to nursing undergraduates with the experience of simulated practice training at certain universities in Seoul city who agreed to participate in the study, suggesting that the study results should not be used for generalization. In addition, there are limitations to defining the correlation in that a cross-sectional study method was used in which the cause and outcome variables of the collected data were measured at the same time point for analysis.

## 5. Conclusions

This study was conducted to identify the relationship between the self-leadership, goal commitment, critical thinking, and problem-solving abilities of nursing undergraduates with the experience of simulated practice training. The multiple regression analysis results show that the variables of goal commitment and critical thinking had a significant impact on the nursing undergraduates’ problem-solving abilities (F = 23.17, *p* < 0.01), with the explanatory power of 41%. In order to enhance the problem-solving abilities of nursing undergraduates, various education programs which involve discussions with simulated training on practice to demonstrate a step-by-step approach to problematic situations, an establishment of hypothesis, and team cooperation must be developed and implemented [30,31,32]. At this stage, measures are required to encourage students to set up their own goals and enhance goal commitment; the effectiveness is expected to increase if the process involves self-leadership. In addition, there may be synergetic effects from students participating in various programs, through training and classes, and developing questions and critical thinking as well as curiosity. Based on the study results, the following suggestions are made. There is a need for repetitive studies on nursing undergraduates and professional nurses under various circumstances and on self-leadership, which was explained as a parameter for problem-solving skills. In addition, a comprehensive program must be developed in which simulated practice training involves not only enhancing the learners’ problem-solving abilities and critical thinking, but also developing self-leadership and goal commitment.

## Figures and Tables

**Table 1:** Mean of Dependent and Independent Variables (N = 260).

Characteristics	M ± SD
Self-leadership	
Behavior-focused strategies	3.67 ± 0.46
Natural reward strategies	3.37 ± 0.59
Constructive thought pattern strategies	3.52 ± 0.60
Goal commitment	3.68 ± 0.57
Critical thinking disposition	
Healthy skepticism	3.60 ± 0.60
Intellectual fairness	3.79 ± 0.55
Objectivity	3.98 ± 0.48
Systematicity	3.32 ± 0.61
Prudence	3.28 ± 0.66
Intellectual eagerness/curiosity	3.46 ± 0.62
Self-confidence	3.59 ± 0.51
Problem-solving abilities	
Positive problem orientation,	3.78 ± 0.52
Negative problem orientation	2.95 ± 0.71
Rational problem solving	3.56 ± 0.45
Impulsive careless style	2.72 ± 0.58
Avoidance coping style	2.68 ± 0.66

M = Mean, SD = Standard Deviation

**Table 2:** General Characteristics of Subjects’ Problem-solving Abilities (N = 260).

Characteristics	n (%)	Problem-Solving Abilities				
		^§^ PPO M ± SD	^‖^ NPO M ± SD	^¶^ RPS M ± SD	^#^ ICS M ± SD	** ACS M ± SD
University						
A	100 (38.5)	3.78 ± 0.49	2.90 ± 0.77	3.55 ± 0.41	2.68 ± 0.58	2.70 ± 0.66
B	56 (21.5)	3.82 ± 0.44	2.92 ± 0.68	3.62 ± 0.44	2.64 ± 0.60	2.64 ± 0.73
C	59 (22.7)	3.71 ± 0.55	2.99 ± 0.65	3.52 ± 0.38	2.78 ± 0.57	2.71 ± 0.64
D	45 (17.3)	3.79 ± 0.63	3.05 ± 0.69	3.58 ± 0.61	2.85 ± 0.55	2.68 ± 0.63
F		0.46	0.53	0.50	1.49	0.14
*p*		0.705	0.657	0.682	0.217	0.933
Age (years)						
≦22	123 (47.3)	3.75 ± 0.55	3.05 ± 0.71	3.53 ± 0.50	2.76 ± 0.59	2.71 ± 0.67
≧23	137 (52.7)	3.80 ± 0.48	2.86 ± 0.71	3.60 ± 0.40	2.69 ± 0.57	2.66 ± 0.65
t		−0.72	2.09	−1.23	0.98	0.66
*p*		0.120	0.916	0.074	0.664	0.964
Grade						
University 3rd	188 (72.3)	3.77 ± 0.55	3.00 ± 0.71	3.56 ± 0.49	2.75 ± 0.62	2.69 ± 0.69
University 4th	72 (27.7)	3.80 ± 0.40	2.80 ± 0.70	3.56 ± 0.34	2.62 ± 0.46	2.67 ± 0.58
T		−0.55	2.05	0.00	1.30	0.24
*p*		0.013	0.866	0.039	0.003	0.213
Religion						
Yes	122 (46.9)	3.82 ± 0.54	2.86 ± 0.69	3.63 ± 0.45	2.64 ± 0.59	2.59 ± 0.64
No	138 (53.1)	3.74 ± 0.49	3.03 ± 0.73	3.50 ± 0.44	2.79 ± 0.56	2.77 ± 0.67
T		1.27	−1.85	2.31	−2.04	−2.10
*p*		0.131	0.697	0.688	0.672	0.343
Interpersonal						
Usually	81 (31.2)	3.65 ± 0.53 ^a^	3.09 ± 0.66 ^a^	3.48 ± 0.49	2.81 ± 0.51	2.74 ± 0.67
Good	147 (56.5)	3.80 ± 0.49 ^ab^	2.97 ± 0.71 ^a^	3.59 ± 0.43	2.71 ± 0.60	2.68 ± 0.67
Very Good	32 (12.3)	4.00 ± 0.51 ^b^	2.51 ± 0.72 ^b^	3.65 ± 0.44	2.55 ± 0.60	2.53 ± 0.57
F		5.68	7.96	2.24	2.34	1.18
*p*		0.004	<0.001	0.108	0.098	0.306
Academic achievement						
≥4.0	32 (12.3)	3.78 ± 0.45	2.96 ± 0.78 ^b^	3.52 ± 0.43	2.59 ± 0.45 ^a^	2.72 ± 0.66 ^a^
3.0–3.9	214 (82.3)	3.78 ± 0.53	2.91 ± 0.70 ^a^	3.58 ± 0.46	2.72 ± 0.59 ^a^	2.65 ± 0.65 ^a^
≦2.9	13 (5.0)	3.70 ± 0.41	3.47 ± 0.64 ^a^	3.40 ± 0.42	3.11 ± 0.48 ^b^	3.21 ± 0.59 ^b^
F		0.13	3.77	1.20	3.78	4.65
*p* *		0.870	0.024	0.302	0.024	0.010
Major Satisfaction						
Unsatisfactory	22 (8.5)	3.59 ± 0.73 ^a^	3.34 ± 0.56 ^a^	3.48 ± 0.69 ^a^	2.95 ± 0.50 ^a^	2.75 ± 0.67
Usually	70 (26.9)	3.62 ± 0.48 ^a^	3.18 ± 0.68 ^ab^	3.43 ± 0.40 ^a^	2.89 ± 0.65 ^ab^	2.89 ± 0.73
Satisfactory	144 (55.4)	3.83 ± 0.47 ^ab^	2.84 ± 0.69 ^bc^	3.60 ± 0.42 ^ab^	2.65 ± 0.51	2.60 ± 0.59
Very satisfactory	24 (9.2)	4.10 ± 0.47 ^b^	2.59 ± 0.76 ^c^	3.82 ± 0.36 ^b^	2.45 ± 0.63 ^b^	2.56 ± 0.73
F		5.35	6.32	5.10	4.24	2.99
*p*		<0.001	<0.001	0.001	0.002	0.019
Leadership training experience						
Yes	99 (38.1)	3.83 ± 0.53	2.82 ± 0.70	3.65 ± 0.38	2.61 ± 0.55	2.59 ± 0.70
NO	161 (61.9)	3.75 ± 0.51	3.03 ± 0.71	3.51 ± 0.48	2.79 ± 0.59	2.74 ± 0.63
T		1.267	−2.32	2.44	−2.34	−1.74
*p*		0.663	0.849	0.212	0.37	0.318
Leadership training needs						
Yes	161 (61.9)	3.79 ± 0.50	2.94 ± 0.72	3.58 ± 0.42	2.73 ± 0.57	2.69 ± 0.66
No	192 (73.8)	3.74 ± 0.57	2.96 ± 0.71	3.52 ± 0.53	2.71 ± 0.59	2.67 ± 0.66
T		0.60	−0.15	0.87	0.14	0.18
*p*		0.275	0.860	0.538	0.858	0.449

^§^ PPO = Positive Problem Orientation, ^‖^ NPO = Negative Problem Orientation, ^¶^ RPS Rational Problem Solving, ^#^ ICS = Impulsive Careless Style, ** ACS = Avoidance Coping Style, * = Fisher’s exact test. ^a,b,c^ = scheffe

**Table 3:** Correlations Between Problem-solving abilities and Other Variables in Participants (N = 260).

	1	2	3	4	5	6	7	8	9	10	11	12	13
1	1												
2	0.43 *	1											
3	0.47 *	0.35 *	1										
4	0.54 *	0.60 *	0.35 *	1									
5	−0.06	0.01	−0.00	0.05	1								
6	0.13 *	0.03	0.06	0.05	−0.31 *	1							
7	0.08	0.05	−0.05	0.03	−0.56 *	0.34 *	1						
8	−0.16 *	−0.15 *	−0.03	−0.08	0.06	−0.04	−0.03 *	1					
9	0.21 *	0.20 *	0.13 *	0.25 *	−0.04	−0.10	−0.04 *	−0.17 *	1				
10	−0.09	0.01	−0.08	0.01	0.06	−0.03	−0.16	−0.05	0.11	1			
11	0.29	0.36	0.24 *	0.29 *	−0.29 *	0.20 *	0.22 *	−0.04 *	0.29 *	−0.09 *	1		
12	−0.16 *	−0.15 *	−0.12 *	−0.13 *	0.08	0.03	−0.15 *	0.05	0.02 *	0.01 *	0.00	1	
13	−0.01	−0.19 *	−0.07	−0.05	0.05	−0.02	−0.03	0.06 *	−0.07	0.04	−0.17	0.10	1

* *p* < 0.05. 1. Problem Solving Skills, 2. Self-Leadership, 3. Goal Commitment, 4. Critical Thinking, 5. University, 6. Age, 7. Grade, 8. Religion, 9. Interpersonal, 10. Academic Achievement, 11. Major Satisfaction, 12. Leadership Training Experience, 13. Leadership Training Needs.

**Table 4:** The Influencing Factors on the Problem-Solving Abilities (N = 260).

Variable	B	SE	β	t	*p*	Tolerance	VIF
Self-Leadership	0.24	0.31	0.05	0.78	0.428	0.56	1.75
Goal Commitment	1.09	0.20	0.28	5.41	0.001	0.82	1.20
Critical Thinking	2.08	0.36	0.36	5.76	0.001	0.59	1.68
Age	0.07	0.05	0.07	1.38	0.166	0.92	1.08
Religion	0.46	0.21	0.10	2.13	0.034	0.94	1.05
Interpersonal	0.02	0.24	0.00	0.08	0.932	0.87	1.13
Major Satisfaction	0.51	0.24	0.11	2.09	0.038	0.78	1.25
Leadership Training Experience	−0.33	0.22	−0.07	−1.50	0.135	0.95	1.04
R = 0.65, R^2^ = 0.43, Adjusted R^2^ = 0.41 F = 23.17, *p* < 0.01, Durbin–Watson = 1.97							

SE = Standard Error, VIF = Variance Inflation Factor.

## Data Availability

The data presented in this study are available on request from the corresponding author.
